# Multimodal fusion models for pulmonary embolism mortality prediction

**DOI:** 10.1038/s41598-023-34303-8

**Published:** 2023-05-09

**Authors:** Noa Cahan, Eyal Klang, Edith M. Marom, Shelly Soffer, Yiftach Barash, Evyatar Burshtein, Eli Konen, Hayit Greenspan

**Affiliations:** 1 Department of Biomedical Engineering, Tel-Aviv University, Tel Aviv, Israel; 2 Department of Diagnostic Imaging, Sheba Medical Center, Ramat Gan, Israel affiliated with the Tel Aviv University, Tel Aviv, Israel; 3 Biomedical Engineering and Imaging Institute, Radiology Dept., Icahn School of Medicine at Mount Sinai, New York, United States

**Keywords:** Cardiovascular diseases, Haematological diseases, Disease-free survival, Risk factors, Machine learning, Image processing, Predictive medicine

## Abstract

Pulmonary embolism (PE) is a common, life threatening cardiovascular emergency. Risk stratification is one of the core principles of acute PE management and determines the choice of diagnostic and therapeutic strategies. In routine clinical practice, clinicians rely on the patient’s electronic health record (EHR) to provide a context for their medical imaging interpretation. Most deep learning models for radiology applications only consider pixel-value information without the clinical context. Only a few integrate both clinical and imaging data. In this work, we develop and compare multimodal fusion models that can utilize multimodal data by combining both volumetric pixel data and clinical patient data for automatic risk stratification of PE. Our best performing model is an intermediate fusion model that incorporates both bilinear attention and TabNet, and can be trained in an end-to-end manner. The results show that multimodality boosts performance by up to 14% with an area under the curve (AUC) of 0.96 for assessing PE severity, with a sensitivity of 90% and specificity of 94%, thus pointing to the value of using multimodal data to automatically assess PE severity.

## Introduction

PE is associated with significant morbidity and mortality and accounts for more than 100,000 annual deaths in the United States^1,2^. Early identification and prompt treatment can greatly improve outcomes. Immediate reliable risk stratification constitutes the cornerstone of PE management, because it can identify patients with a higher risk of death who need specific interventions, or alternatively, those suitable for outpatient management^1,3^. The key to effective treatment of acute PE lies in the assessment of the patient’s early death risk. Thirty-day all-cause mortality is the most common measure of prognosis^4^.Available tools for PE prognosis assessment include (1) Imaging tests such as Echocardiography (Echo) and computed-tomography pulmonary angiography (CTPA); (2) Clinical prediction scales such as the Pulmonary Embolism Severity Index (PESI)^5^ and the simplified PESI (sPESI)^6^; and (3) Laboratory markers such as BNP, NT-proBNP, Cardiac troponin I-T, H-FABP and GDF-15. Currently, no individual laboratory marker or imaging sign has been shown to justify advanced therapy in patients with PE. Therefore, attention is shifting to prognostic models combining clinical, imaging, and biochemical parameters^7^. The task of automated risk stratification of PE still poses a challenge for researchers and clinicians. The variability in the appearance of a PE on imaging, together with the lack of discriminative warning signs or symptoms, make the PE distinction difficult. In the current study, we present an automated solution for the risk stratification of PE based on the patient’s early death risk. We use the thirty-day all-cause mortality label as a measure for PE severity assessment. Throughout the paper the terms PE severity, PE risk stratification and PE thirty-day all-cause mortality prediction are used interchangeably.

Multimodal deep learning models for automated clinical decision support and outcome prediction have been gaining popularity in recent years^8^. For example, demographic data and lab tests have been combined with imaging data for the prediction of Alzheimer’s disease and were shown to be an improvement over single data source models^9–11^. Other studies have reported similar advantages for a wide range of medical applications from radiology^12^ to oncology^13^. For the task of PE detection, much of the current work focuses on imaging only solutions, in which a detection of the actual clot as seen in CTPA scans is the desired outcome^14–18^. In these studies, the PE methods are either fully supervised, where the datasets contain full segmentation maps or are labeled on the 2D slice-level. Promising results for PE detection have been shown based on imaging data alone, and are further improved when combined with additional modalities. Both Huang et al.^19^ and Somani et al.^20^ proposed a multimodal fusion model combining EHR data with CTPA and ECG respectively for the detection task. When designing a multimodal fusion solution, several models can be considered: *Multimodal vision-language models* trained with a contrastive objective^21,22^ have enabled zero-shot adaptation to novel tasks, without the need for fine-tuning. In 2022, Flamingo^23^ was proposed, a Visual Language Model that sets a new state of the art in few-shot learning on a wide range of open-ended vision and language tasks. These models achieve state-of-the-art results when pretrained on an large-scale multimodal corpora, but their ability to perform well with a small dataset is still uncertain. Several prior works have considered the problem of modeling multi-modal data using *generative VAE-inspired models*. JMVAE^24^ exploits the joint inference network to learn the interaction of two modalities. MVAE^25^ considers only partial combination of observed modalities in order to increase the computational efficiency of learning. Both methods learn a single latent space to represent the multi-modal data. In contrast, DMVAE^26^ learns a disjoint private and shared space in a multi-modal setting. The self attention operation of *Transformers*^27^ provides a natural mechanism to connect multimodal signals. Many works have studied Transformers extensively for various multimodal tasks, and shown to be compatible with various modalities in both discriminative and generative tasks^28–31^. Some previous works have used single modality convolutional neural networks (CNN) backbones as input to transformers^32^ , while other models directly combine tokens from different modalities as inputs to the transformers^33^. A noticeable fusion scheme is introduced based on a notion of bottleneck tokens^34^. It applies for both early and middle fusion by simply choosing to-be-fused layers. The use of generative models and Transformers in this study is limited by the need for a large amount of data and more model parameters. The dataset in this work is extremely small and highly imbalanced with an even smaller portion of positive examples making it very difficult to utilize the above methods. Most previous work has focused on approaches using only one of several possible fusion strategies. These have relied on a few manually selected clinical features. The relative performance of different fusion techniques has not yet been explored. Clearly, however, understanding how to facilitate high-level interactions between different modality features is likely to boost model performance.

**Figure 1:**
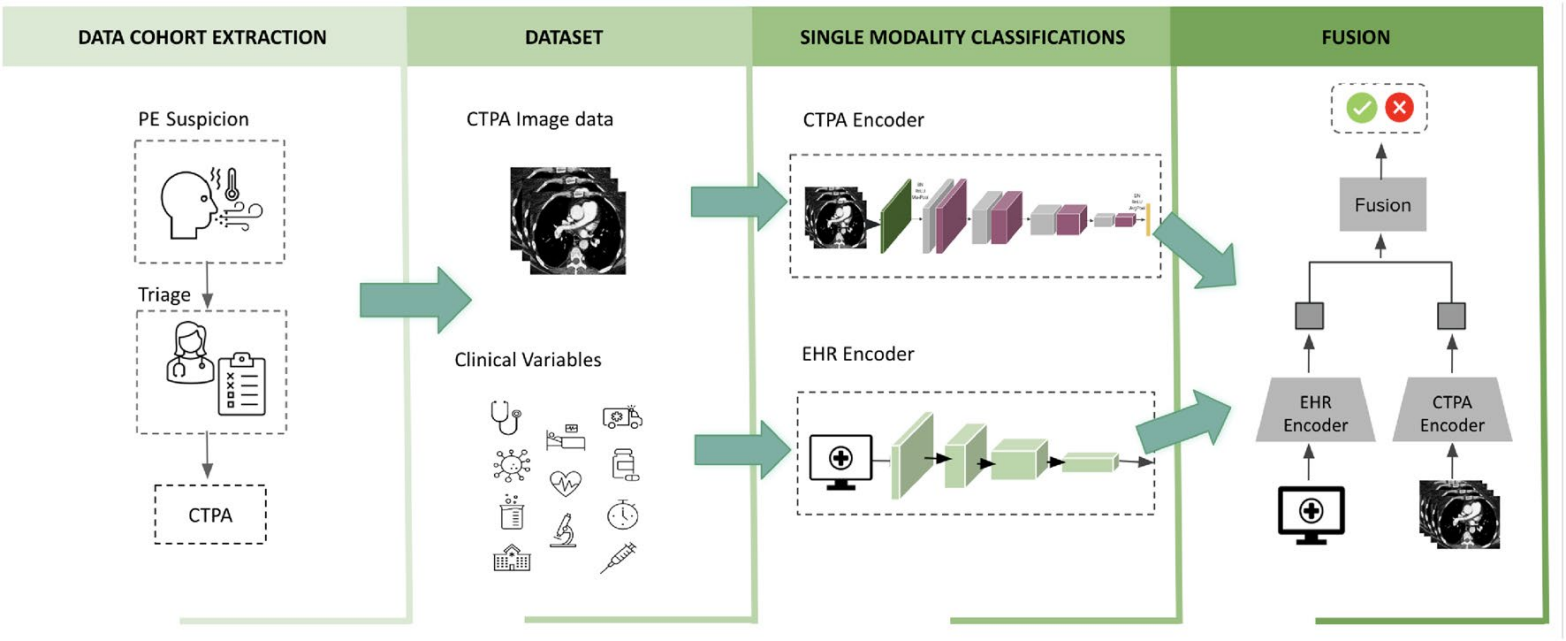
Study design. The first stage was to establish the cohort. Our pipeline for creating models for PE risk stratification consisted of collecting two data modalities: clinical data (EHR) including patient demographics, comorbidities, vital signs, and relevant labs, and CTPA scans. We risk-stratified the diagnosed PE using the 30-day all-cause mortality label from the imaging data and the clinical variable data independently. Finally, these data were linked using the unimodal classifiers from the previous stage to develop, analyze, and benchmark the models for PE prognosis. (Icon images from Microsoft PowerPoint 2023).

In the current study, we addressed some of the gaps mentioned above. Focusing the methodology on the routine clinical practice in PE care, we combined key clinical and laboratory data along with the imaging data. In order to make the proposed models robust and generalizable, we paid special attention to formalizing the solution to a scenario of limited data and limited labeling. Instead of relying on manually produced pixel-level annotations, a single patient-level label was used. Overall we provide a fully automated multimodal fusion network that can be trained in an end-to-end manner without requiring preprocessing or labor-intensive labeling of the data. In this work, we developed and compared diverse multimodal fusion model architectures for risk stratification of PE. The models can handle multimodal data combining (1) CTPA imaging and (2) EHR tabular data on demographics, co-morbidities, vital signs, laboratory results and clinical scores. We tested early, late and intermediate fusion strategies, and compared their performance to better leverage the various data modalities. We combined TabNet^35^, a novel deep neural network (DNN) for tabular data achieving state-of-the-art results, with a convolutional neural network (CNN), which allows for concurrent image and tabular data analysis. In doing so, we extend the use of TabNet (which is commonly implemented as a classifier) to research its contribution as a tabular data *encoder* and as a multimodal attention classifier. In addition, we evaluated the contribution of both single and multimodality classifiers. The results show that this multimodal learning method boosts prediction metrics and outperforms single modality classification networks. To introduce model interpretability and facilitate subsequent clinical applications, we highlight the image regions and tabular features that our model uses for prediction. The technique illustrates how a modest, minimally preprocessed and unmarked dataset of clinical data and CTPAs can be utilized to effectively risk stratify PE. Figure 1 outlines the study design used for this study.

The current work is an extension of our earlier work^36^. The main contributions of the current work can be summarized as follows:

1. *PE risk stratification from multimodality data*. To the best of our knowledge, no previous works provide an overall prognosis for PE treatment, and none combine multimodal data for automatic PE risk stratification.
2. *We present a thorough examination of multimodal fusion models for prognostic tasks*. Leveraging both clinical and imaging data by using a variety of fusion approaches may not only lead to a satisfactory model selection which reduces the misdiagnosis rate and delays in treatment response, but also aids future work by exploring the optimal fusion strategies and data selection.
3. *End-to-end solution with minimal data annotations*. The dataset is labeled with a single binary label. Specifically, the whole 3D-CTPA scan is annotated using one scan-level label, with no additional markings or even any slice-level annotation. Our approach provides for a completely automated network in which the entire volumetric scan and tabular data are fed to the network with only minimal preparation, thus allowing for end-to-end training.
4. *TabNet for multimodality classification*. While gaining in popularity as a classifier for tabular data, TabNet has been much less explored as a tabular data encoder or as a multimodality feature selector and classifier. Our method combines CNNs and TabNet to leverage multimodal features for classification.

## Results

### Model performance

#### Multimodal classification performance against single modality classifiers

We compared the performance of the single modality models with the multimodal classifiers and existing clinical scores. The unimodal classifiers were used as our baseline. Table 1 summarizes the results. On the holdout test set, our best fusion model significantly outperformed both of our top single modality models with an AUC of 0.96 [95% CI: 0.93–1.0], followed by the EHR model (AUC 0.87 [95% CI: 0.78–0.95]), and the CTPA model (AUC 0.82 [95% CI: 0.74–0.91]). It is evident that fusing different data modalities enhanced performance compared to using only a single data modality. When comparing the two CTPA baselines, the CNN-based SANet outperformed the Swin UNETR Transformer with a gain of 9% in AUC. The intermediate model trained with a Swin UNETR backbone achieved similar results to the best multimodal classifier with no statistical significant difference.

In this study, we set our operating point based on the Youden’s J-Score statistic that maximizes the sum of sensitivity and specificity on the validation set. For the numerical threshold that separates the predicted classes, we utilized the standard definition of the operating point. By applying this threshold, the model achieved a sensitivity of 90%, a PPV of 69% and a specificity of 94% when predicting 30-day mortality. Applications in clinical settings, however, are sometimes tuned to maximize sensitivity in order to minimize the false-negative rate. We were able to further improve the fusion model’s sensitivity at the cost of lowering the PPV: in this case the fusion model achieved a sensitivity of 1.00, a specificity of 0.90 and a PPV of 0.625 across the test set.

**Table 1:** Comparing the AUC scores (with a 95% confidence interval) of the multimodal classifiers to single modality classifiers and existing clinical scores.

Model	AUC	Accuracy	Specificity	Sensitivity	PPV	NPV
**Single modalities**						
EHR	0.87 [0.78–0.95]	0.81	0.79	0.9	0.41	0.98
CTPA-SANet	0.82 [0.74–0.91]	0.79	0.79	0.8	0.38	0.96
CTPA-Swin UNETR	0.73 [0.6–0.85]	0.56	0.52	0.8	0.21	0.94
**Clinical predictive rules**						
PESI	0.8 [0.7–0.87]	0.61	0.56	0.9	0.25	0.97
sPESI	0.77 [0.63–0.87]	0.48	0.42	0.9	0.20	0.96
**Fusion models**						
Late-average (Fig. 7a)	0.85 [0.75–0.93]	0.68	0.65	0.9	0.29	0.98
Late-TabNet (Fig. 7b)	0.92 [0.87–0.98]	0.89	0.89	0.9	0.56	0.98
Late-XGBoost (Fig. 7c)	0.88 [0.8–0.95]	0.76	0.74	0.9	0.36	0.98
Early (Fig. 7d)	0.9 [0.83–0.96]	0.81	0.79	0.9	0.41	0.98
Intermediate + SANet(Fig. 7e)	${\textbf {0.96 [0.93-1.0]}}$	${\textbf {0.93}}$	${\textbf {0.94}}$	${\textbf {0.9}}$	${\textbf {0.69}}$	${\textbf {0.98}}$
Intermediate + Swin UNETR (Fig. 7f)	0.95 [0.9–0.98]	0.87	0.87	0.9	0.53	0.98
MBT (Fig. 7g)	0.9 [0.85–0.96]	0.82	0.81	0.9	0.43	0.98

The intermediate fusion model significantly outperformed all the other methods (Kolmogorov-Smirnov test, with $p \le 0.05$).

Best performing values are in [bold].

#### Multimodal classification performance vs. existing clinical scores

**Figure 2:**
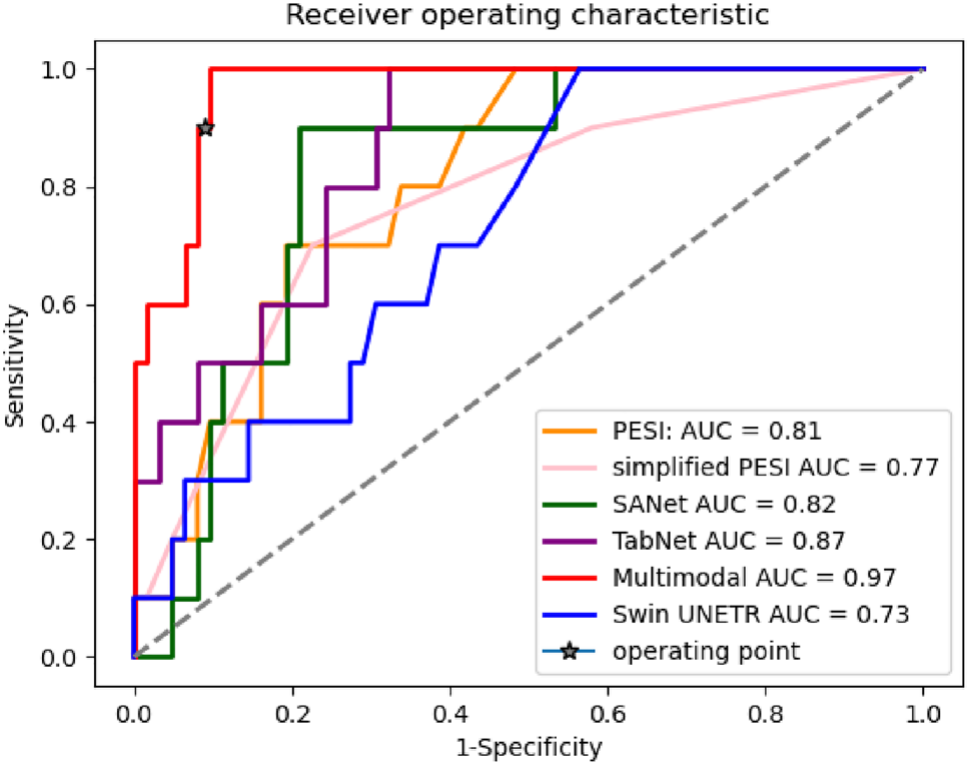
Comparison of AUC plots between single modality classification baselines, our leading multimodal classifier and existing clinical predictive rules.

We also compared our results to existing clinical scores. According to the European Society of Cardiology (ESC) guidelines, the best-known standards are PESI and sPESI. They were used here to identify low-risk patients who could safely be treated as outpatients^37^. The PESI and sPESI calculated scores for our dataset match the reported AUC and sensitivity in literature. As can be seen from the results (Table 1, middle), despite the high sensitivity and an NPV that was similar to our multimodal classifier, PESI and sPESI lacked specificity to predict early mortality. In contrast, the multimodality classifier presented both a high specificity and sensitivity. Figure  2 provides a visual comparison of the AUC scores for the different baseline models, clinical scores and our top performing multimodal classifier.

### Model fairness

We evaluated the models performance based on patient demographics information (age and gender) to evaluate model fairness. The patient demographics split (age and gender) is presented in Fig. 8. The AUCs of the best performing model and existing clinical scores are presented in Table 2. As can be seen from the results, the existing clinical rules, PESI and sPESI presents a much higher bias in the results compared to our multimodal classifier. The multimodel classifier shows only slight bias. It is anticipated that there may be some bias in the outcomes, particularly with regards to clinical scores, due to age and gender being factors in the scoring.

**Table 2:** Comparing the AUC scores (with a 95% confidence interval) of the top multimodal classifiers and existing clinical scores based on patient demographics information.

Model	Full	Male	Female	Age 0–50	Age 50–70	Age 70–95
Multimodal	0.96 [0.93–1.0]	0.98 [0.94–1.00]	0.97 [0.92–1.00]	0.96 [0.84–1.00]	0.98 [0.93–1.00]	0.95 [0.87–1.00]
PESI	0.8 [0.7–0.87]	0.77 [0.63–0.92]	0.84 [0.67–0.98]	0.92 [0.75–1.00]	0.86 [0.69–1.00]	0.71 [0.42–0.93]
sPESI	0.77 [0.63–0.87]	0.72 [0.5–0.92]	0.84 [0.7–0.95]	0.73 [0.46–0.93]	0.93 [0.86–0.98]	0.6 [0.2–0.89]

### Ablation study on fusion strategies

The performance of each fusion model is detailed in the bottom part of Table 1. Over the entire hold-out test set, the CNN-based intermediate fusion strategy model achieved the highest test AUC of 0.964. When using bootstrapping to compute the p-values between each model, this model outperformed the other fusion architectures significantly. The model outperformed the next best model by a margin of 4% in AUC. All the fusion models improved performance compared to the unimodal classifiers, except for the first model, as shown in Fig. 7a, a late fusion model that combines decisions using a weighted average. This suggests that some non-linear aggregation is necessary to employ the different modalities. We also compare our results to Multimodal Bottleneck Transformer (MBT)^34^. Despite having high potential, MBT failed to outperform the intermediate model.

### Ablation study on architecture choices

Table 3 evaluates several design choices and the contribution of different network components. We examined the importance of TabNet as the final classification element and explored the model’s performance using 1DCNN^38^, XGBoost and a simple fully connected (FC) layer as alternative classifiers. As can be seen from the results, all these methods improved the results compared to single modalities, but TabNet performed best. We also explored the model’s variability when revising the vector size of the extracted embedding from the image and tabular data unimodal classifiers. For the selected model we chose a vector size of 64 and tested it for sizes of 256 and 1024. It is evident that our method was stable to this parameter in terms of the AUC metric. We researched the significance of dimensionality reduction on the concatenated feature vector and explored various algorithms in addition to PCA. This involved testing several matrix factorization methods including the sparse version of PCA^39^ and non-negative matrix factorization (NMF)^40^. We also tested manifold learning methods including Locally Linear Embedding (LLE)^41^ in its modified version and Spectral embedding^42^. Finally, we hypothesized that in addition to finding a shared representation, dimensionality reduction would act similarly to the attention mechanism since it selects the most salient features. We thus replaced the PCA in our model with bilinear attention. Clearly, dimensionality reduction significantly improved performance in all metrics compared to a non-reduced vector. Overall, most of the algorithms tested performed similarly and boosted performance, including bilinear attention. This may imply that bilinear attention can replace other dimensionality reduction techniques.

**Table 3:** Ablations on the intermediate model.

Model	AUC	Accuracy	Specificity	Sensitivity
**Final component architecture**				
FC layer	0.9* [0.82–0.95]	0.89	0.9	0.8
1DCNN	0.91* [0.82–0.95]	0.89	0.89	0.9
XGBoost	0.92* [0.84–0.97]	0.88	0.88	0.9
**Dimensionality reduction**				
w/o	0.92* [0.87–0.97]	0.85	0.84	0.9
Sparse PCA	0.94 [0.88–0.98]	0.89	0.89	0.9
NMF	0.94 [0.88–0.98]	0.85	0.84	0.9
Modified LLE	0.96 [0.92–1.0]	0.9	0.92	0.8
Spectral embedding	0.93* [0.89–0.97]	0.85	0.84	0.9
BiAttention	0.95 [0.9–0.99]	0.9	0.9	0.9
**Embedding dimension**				
d = 1024	0.95 [0.9–0.98]	0.89	0.89	0.9
d = 256	0.96 [0.93–1.0]	0.92	0.92	0.9
Full	${\textbf {0.96 [0.93-1.0]}}$	${\textbf {0.93}}$	${\textbf {0.94}}$	${\textbf {0.9}}$

Asterisks mark statistically significant difference compared to the full intermediate model architecture (Kolmogorov-Smirnov test with $p \le 0.05$ ).

Best performing values are in [bold].

### Latent space feature visualization

**Figure 3:**
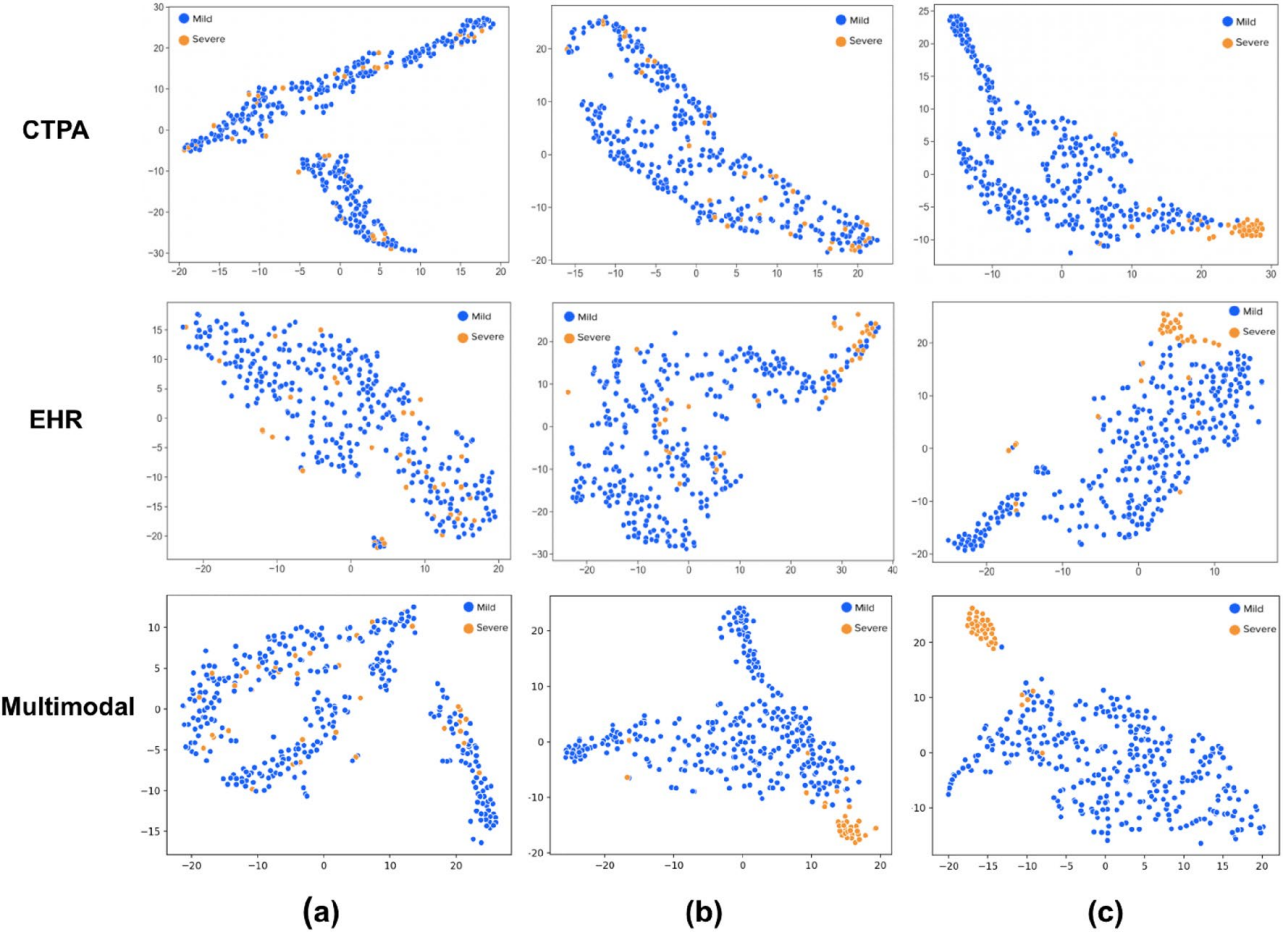
The t-SNE visualization of the progress of the latent space embeddings during the training process for the single CTPA and EHR data modalities (top and middle rows respectively) and multimodality (bottom row). Features were extracted from the start (after only one epoch), middle and end of the training process (columns (**a**–**c**) respectively.). The multimodality features were extracted from the multimodal classifier and formed 128-d vectors, which were then embedded in the 2D space by t-SNE for visualization. The extracted features from each single modality classifier were of size 64-d. (**c**) The final trained embeddings are presented. For both single and multimodal classifiers the two groups of samples are well-separated in the 2D space. It is evident that the multimodal classifier performs better sample separation and earlier on in the training process.

The progression of the multimodality features in the embedded space during the training process is presented in Fig. 3. The embeddings were extracted from the intermediate model (Fig. 7e) and were constructed from the concatenated features from each unimodal model. In order to visualize the high-dimensional features, we employed t-SNE^43^ on the concatenated 128-d embedded vector. The two groups of samples are color-coded, where positive is orange and negative is blue. The figure depicts different phases in the training process. In Fig. 3a, the embeddings were extracted after one epoch of training thus making the two classes hard to categorize. Figure 3b shows the features extracted from the trained unimodal classifiers and after dimensionality reduction. This explains why the two classes were somewhat distinct but still contained errors. Figure 3c shows the embeddings after training the final TabNet block. As can be seen, the two classes can clearly be separated for classification with minimal outliers.

### Interpretation of the model predictions

#### Imaging

Our best performing model was the intermediate model shown in Fig. 7e. As part of the model’s pipeline we applied PCA to the concatenated unimodal vectors, which is a non-differential algorithm. In order to obtain model interpretability, we replaced the PCA with a bilinear attention mechanism. We showed in the architecture choices ablation study section that this variation on the model’s architecture achieved similar performance metrics. In addition, this change made the model differential which allowed us to use gradient back-propagation techniques for model interpretability.

**Figure 4:**
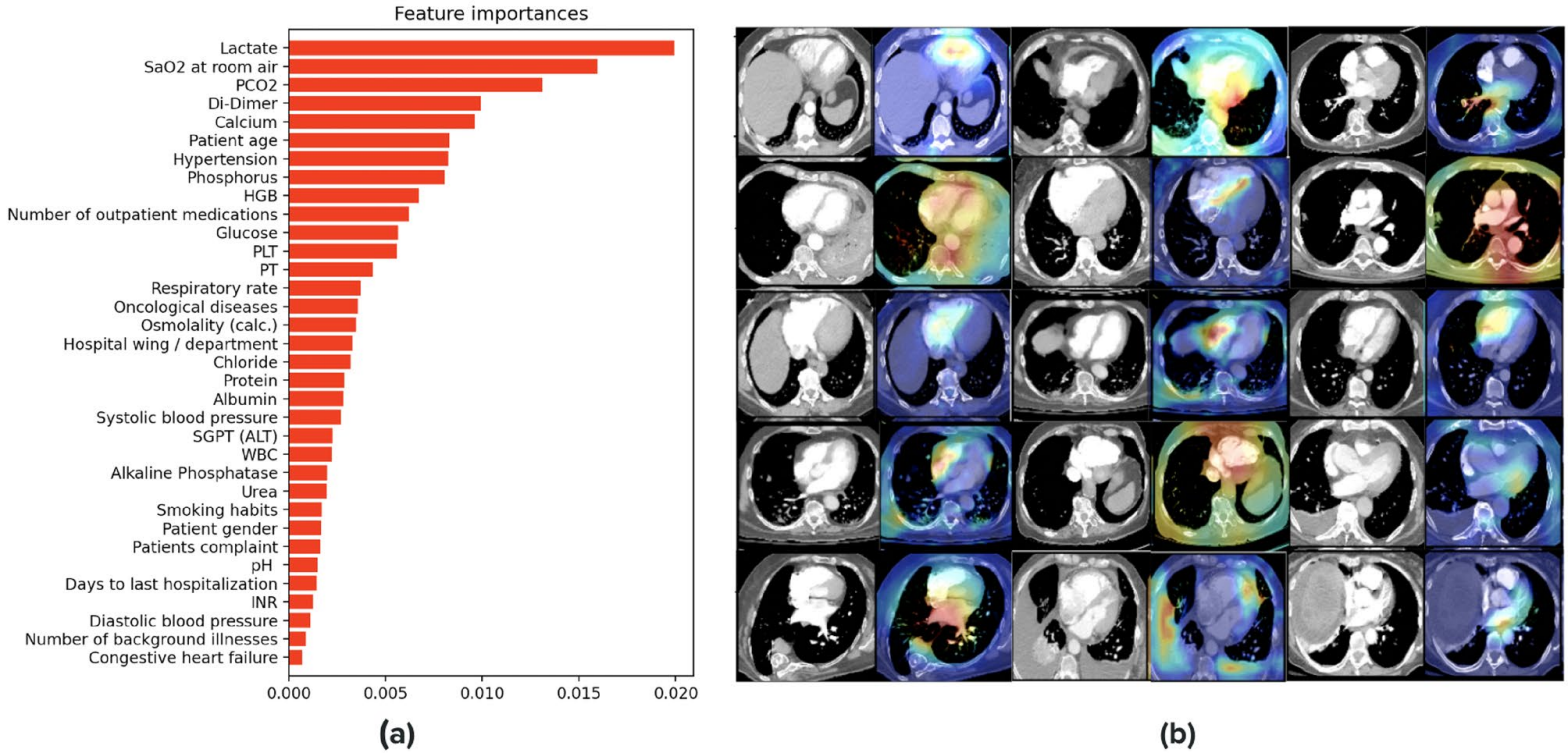
Interpretation of the model predictions for the image and EHR modalities: (**a**) EHR selected feature set from the multimodality classifier. The features presented are the average over the test set. (**b**) Grad-Cam visualization results of correctly classified examples: pairs of 2D slices taken from 3D CTPA scans and their corresponding heatmap results. High activations (red, yellow and green) indicate the focus areas that the model selected for classification. Note the high activations in anatomically relevant regions such as the pulmonary trunk, RV and LV chamber, aorta, IVC and heart area.

We highlighted the features selected by the model during prediction and identified locations and slices in the 3D-CTPA imaging scan that contributed the most to the classification using 3D gradient-weighted class activation mapping (Grad-CAMs)^44^. Figure 4b illustrates the heatmap visualization results obtained from our network. The model contains two different inputs: 3D-CTPA imaging scans and EHR tabular data. The visualization results were created by back-propagating through the imaging part of the model. The brightest areas of the heatmap are regions that influenced the model prediction the most.

We anticipated that during prediction, the model would select descriptors correlated with high risk PE which are located around the heart area as well as the hepatic veins. As expected, the network focused on the pulmonary artery and trunk as well as the aorta, the heart area (specifically the right and left ventricle (RV, LV) chamber) and the inferior vena cava (IVC). Interestingly, the model also picked up on the superior vena cava (SVC) which is more challenging for radiologists to view on CTPAs. We note that the Grad-CAM predictions mostly do not or only weakly cover the PE ground truth of the scans. This fact supports acute PE severity assessment literature which indicates that the extent of the PE clot on CT does not always correlate with the clinical severity of acute PE or its impact on RV function^7^. Figure 4b depicts some of these instances.

#### EHR data

To interpret the EMR data part of the model, we used TabNet’s feature importance masks. The mask aggregates the attention maps used in each step of the model for feature selection. These masks are weighted and summed, thus providing each feature with a gain as an indicator of feature importance. Figure 4a lists a subset of the features with the highest gain.

As expected, the tabular data associated with high-risk PE included either features representing short-term acuity or features representing a chronic debilitating condition. The short-term features included vital signs such as blood pressure, saturation and respiratory rate, and acute phase laboratory results such as blood gases, lactate and WBC. The long-term features included age at presentation, co-morbidities and home medication.

#### Misclassified examples

**Figure 5:**
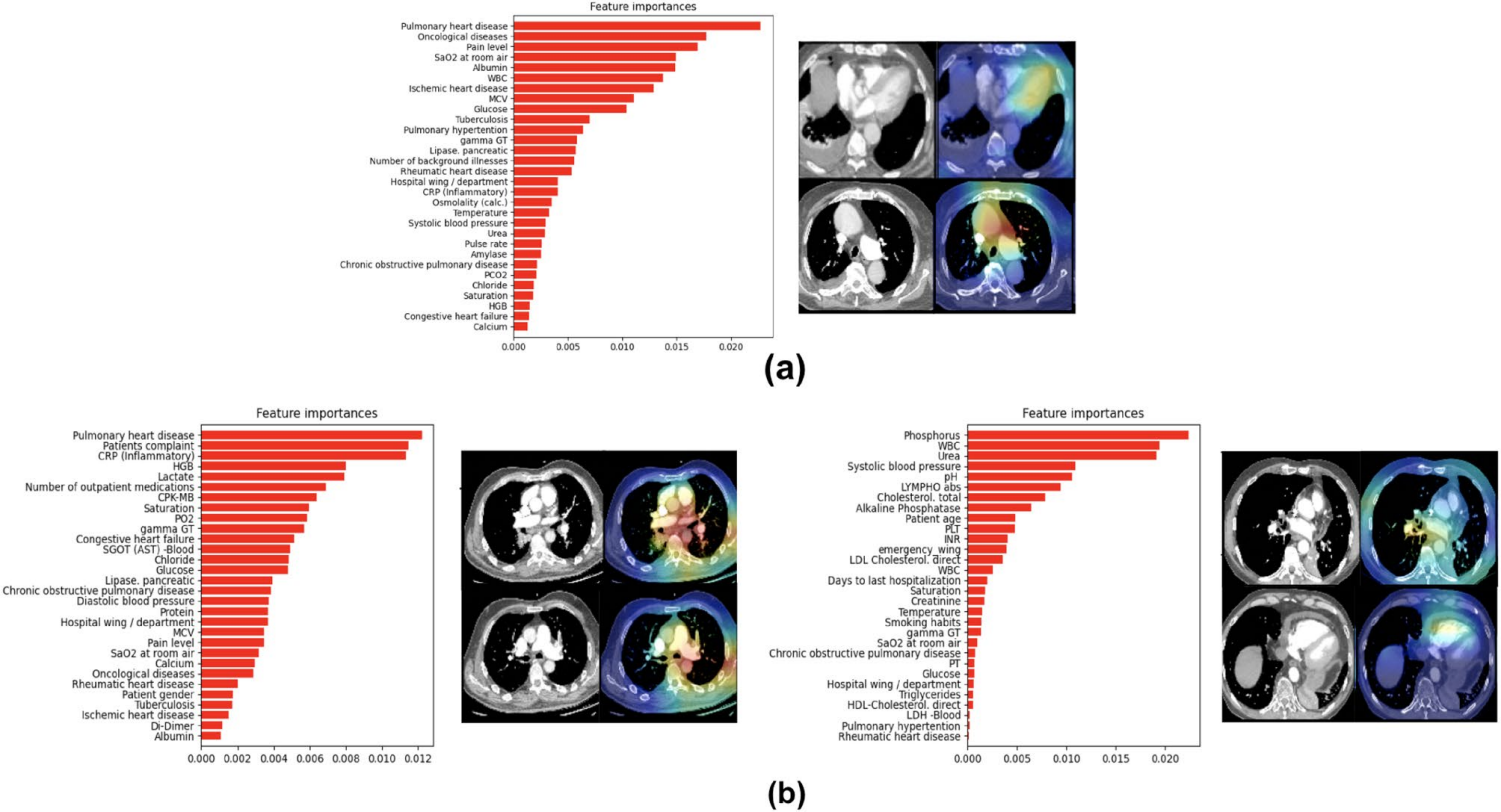
Misclassified examples: (**a**) False negative example. (**b**) Two false positive examples. For each example, the EHR selected features from the multimodal classifier arrange by importance gain are presented on the left. On the right—representative Grad-CAM visualizations. (**a**) The heatmaps overlay the heart area, specifically the LV and also the pulmonary artery (PA). The EHR features indicate a number of illnesses that support this such as pulmonary hypertension and other heart conditions. (**b**) The false positive examples indicate acute PE and overall severe illness as well focusing on the pulmonary truck, Aorta (left), RV chamber and also covering the PE clot (right) with EHR feature supporting this.

In this section we analyze one false positive (FP) and two false negative (FN) examples (Top and bottom of Fig. 5 respectively). Our analysis is limited as we have only one FN example and very few FP examples in our test set. Both the FP and FN examples illustrate individuals who are suffering from acute PE and experience a general state of severe condition. The heart and pulmonary trunk area is enlarged and the characteristics of the EHR suggest the presence of several illnesses, including pulmonary hypertension and various heart ailments. It is noteworthy to observe these mislabeled instances, particularly the FPs, as a physician would probably classify them in the same manner as the model.

## Discussion

The purpose of this study was to build a multimodal deep learning model that could successfully leverage information from both volumetric CTPA scans and tabular EHR data for PE risk stratification. The network was trained in an end-to-end manner, from a single label of 30-day all-cause mortality. We developed and evaluated different multimodal deep learning models that enabled us to make use of the different data modalities in an optimal manner. Our best performing model was an intermediate fusion model using 3D CNN and TabNet. The fusion model achieved a state-of-the-art AUC of 96% and improved the PE severity classification by 14% and 9% over the image-based and EHR-based models, respectively.

The success of the presented model can be attributed to a number of factors: (1) The ability to create meaningful embeddings for various data modalities. In particular, the use of TabNet as the EHR data encoder is novel. In general, deep learning architectures are not well-suited for tabular data and fail to find optimal solutions for tabular decision manifolds^45,46^. The typical strategy for EHR data is to apply decision tree architectures. Despite performing well, they do not have data encoding capabilities. (2) As the final multimodal classification component in our architecture, TabNet performed best. (3) The application of dimensionality reduction on the concatenated single modalities vector, whether performed using bilinear attention or a different algorithm, provided an additional boost in performance since it could exploit the interactions between the two groups of data modalities while extracting their joint representations.

While Vision and Multimodal Transformers showed state-of-the-art results when trained on large corpora^23,47^, our study found that both the CTPA Swin UNETR model and the multimodal MBT were surpassed by CNN models. Transformers require a vast amount of data for training due to their large model parameter capacity^48^, which may explain the difficulty in our study considering our relatively small dataset. The intermediate model trained with Swin UNETR backbone achieved similar results to the best multimodal classifier, indicating that the backbone did in fact learn a meaningful representation of the scan.

Despite their highly appealing ability to handle missing modalities^24^, we did not consider exploring generative VAEs for our multimodal classifier, with similar concerns as with transformers. We believed that our small dataset may struggle with multimodal VAEs which typically requires a substantial amount of data to produce high-resolution reconstructions, especially due to the fact that our data would need to be processed in a 3D manner. For example, both Litany et al.^49^ and Tan et al.^50^, successfully used a VAE for 3D mesh reconstruction but were trained on around 20k examples. Multimodal motion and speech audio of the speaker incorporates VQ-VAE in a notable manner but used 72 hours of recorded data^51^. Even in^52^, a StyleGAN classifier which was specifically designed to perform well using extremely small annotated datasets, the self-supervised training process still utilized a large amount of unlabeled data. These examples support our concern for the need of a large dataset for generative model training. Future studies could explore ways to optimize the use of generative models with smaller datasets, or to develop more efficient algorithms that can handle larger datasets and more complex models.

In this work we explored the use of bilinear attention algorithm for dimensionality reduction in fusion models. Unlike dimensionality reduction algorithms, attention is differentiable, allowing gradient back-propagation and thus end-to-end training. In addition, the Grad-Cam interpretability method relies on back-propagation, which can only be attained in an all-differentiable model.

We compared our results to PESI and sPESI, the most common prognostic scores. Our conclusions also apply to other available prognostic models. While they are sensitive when predicting death from acute PE, they are not specific. One study of 11 clinical prognostic models reported that although the sensitivity of some models, including PESI and sPESI, were > 89%, none had a specificity greater than 48%. There are several clinical prediction rules that predict early mortality in patients with acute PE, but not all of them possess the level of sensitivity necessary to provide clinicians with adequate reassurance^53^. Moreover, these scores rely heavily on demographic and co-morbid conditions rather than on the severity of the acute PE event. In contrast, the multimodality classifier presents both high specificity and sensitivity and may therefore be used as an alternative prognostic model in a clinical setting.

This study is subject to a few significant limitations. It was designed as a single-center, retrospective study, which is associated with well-known shortcomings and inherent limitations. Generalizing the methods and results of our study to other predictive tasks with different modalities should be examined carefully, as our comparison of various fusion types was limited to predicting the severity of PE using CTPA scans and EHR data. Finally, when comparing the tabular and image single modality classifiers it is apparent that the tabular data withheld more features that correlate highly with mortality within 30 days. Considering that most of the patients in this study had background illnesses and pre-existing or concurrent medical conditions, this also may suggest that some of the patients who died in this study died *with* PE rather than *from* PE. This is supported and presented in our analysis of misclassified examples where in both types of misclassifications ( FN and FP) the overall clinical condition of the patient is severe and it is unclear why the system chose one decision over the other. Finally, our model lacks the ability to run effectively in the absence of a modal data. In future work, we intend to explore a more flexible architecture that will allow this.

Machine learning models that consider both CTPA scans and EHR data as do radiologists, offer better prognosis designations over imaging data alone. Multimodal data fusion models may improve the clinical utility of automating medical imaging tasks and are well-suited for adoption in clinical practice. This fusion model may be more optimal for integration into the clinical workflow given its end-to-end approach and high sensitivity, which helps reduce false-positives and clinical alert fatigue.

## Methods

This section presents the multimodality fusion strategies and models for the task of PE risk stratification. In order to demonstrate the effect of multimodality fusion, we first introduce the single modality encoders that were used as the baseline classification models for comparison and as single modality feature extractors that were applied for fusion in the multimodality setting.

### Unimodal classification models

#### Clinical variable encoder

Clinical variables taken from the patients’ EHRs include demographics, vital signs, chief complaint, clinical history, inpatient and outpatient medications, past medical history (ICD-9 codes) and laboratory test results. Additional variables included presenting time (year, month, day, hour) escort type and the ED wing where the patient was triaged. The demographic features consisted of gender, smoking habits and age. For vitals, we included systolic and diastolic blood pressure, fever, respiration rate, saturation and heart rate. Clinical history included previous ED visits and hospitalizations as well as known co-morbidities. More than six hundred unique classes of drugs were identified for inpatient and outpatient medication. Each medication was represented as a binary label indicating whether the drug was prescribed to the patient. The number of drugs per patient was added as a variable as well. We only kept frequently assigned ICD-9 codes with more than 5% occurrences in the training dataset, for a total of 308 diagnoses. We used a binary label indicating presence/absence. Finally, we identified over 40 laboratory blood tests and kept their latest measurement. Each category of the tabular data was parsed and feature-engineered differently.

We explored two machine learning methods to classify the clinical data. For the first model, we applied a gradient boosting decision tree algorithm. Specifically, we used *XGBoost*^54^ to classify PE severity using all the clinical features. The main strength of decision trees lies in selecting global features that are the most statistically informative^55^ and are commonly used for tabular data learning. Currently, XGBoost provides state-of-the-art results in many applications and dominates most of the recent data science competitions.

The second model executed *TabNet*^35^, a deep learning model specifically designed for raw tabular data inputs that achieves state-of-the-art results. It uses sequential attention for sparse feature selection and mimics a gradient boosting decision tree algorithm in a neural network setting. It is trained using gradient descent-based for end-to-end learning. Alternative attempts to use deep neural networks have failed to achieve competitive results. Before feeding into each model, all the input features were normalized by subtracting the mean and dividing by the standard deviation.

#### CTPA imaging encoder

The imaging part of our dataset was composed of a contrast-enhanced chest CT series. Digital Imaging and Communications in Medicine (DICOM) scans were extracted. The DICOM scans were resampled to adjust the spacing of the pixels in each dimension in order to achieve an isotropic voxel size. The resulting cubic pixel array was then resized to size 128 $\times$ 128 $\times$ 128 and saved for each scan. This full 3D scan was fed into the model as a whole.

**Figure 6:**
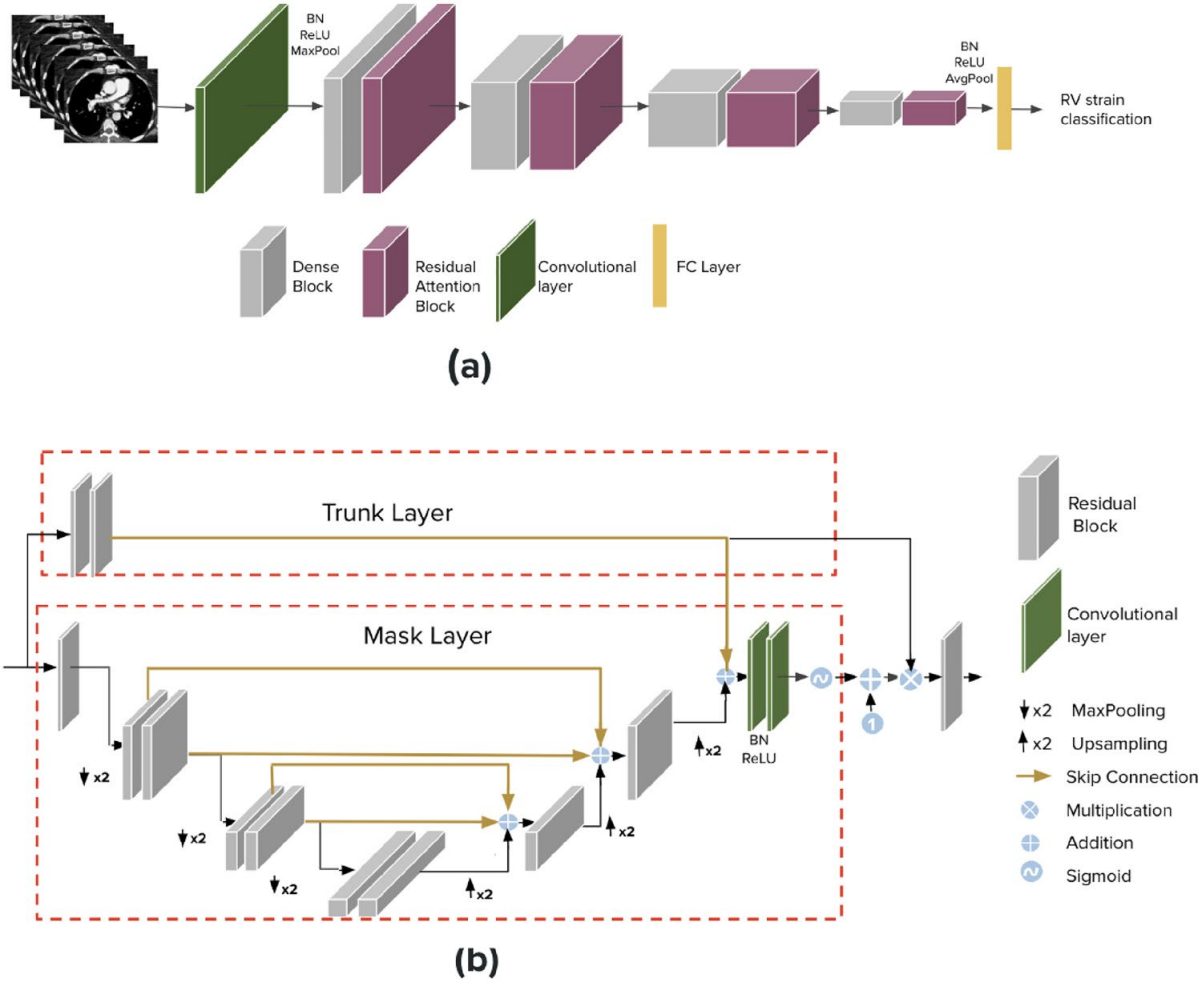
(**a**) CTPA image encoder-SANet. 3D DenseNet with stacked residual attention blocks. (**b**) The attention block architecture.

We examined two different baseline models for the imaging data. The first one is a CNN-based model that we developed in earlier work^56^termed SANet. This network takes 3D input data and utilizes a single binary output label for the task of classification. In^56^, we demonstrated the viability of the network to detect a key finding within the input volumetric data. Specifically, we show that a small dataset of unmarked CTPAs can be used for effective RV strain classification achieved state-of-the-art results. Our developed network, termed SANet, takes as input 3D data and a single binary label for the task of classification. A brief description of SANet follows: The network’s backbone is a 3D DenseNet architecture, further improved by residual attention blocks. The attention blocks are incorporated between the network’s layers as an integral part of it, allowing easy end-to-end training. Figure 6a depicts the block diagram of the developed system. Each attention block is comprised from a mask and trunk units. The trunk branch is constructed from ResNet residual block units to perform feature extraction. The mask is structured from a soft attention structure with an autoencoder design that resembles the popular U-Net^57^ to mask the features extracted from the trunk. Thus, the mask behaves as a feature selector. The block diagram of the attention block can be visualized in Fig. 6b. This design can be trained easily in an end-to-end manner without requiring computationally intensive and time-consuming preprocessing or strenuous labeling of the data. In^56^, we performed extensive analysis and ablation studies comparing SANet to other 3D classification models and chose it as our imaging encoder due to its high performance. The original manuscript provides additional details on the model architecture and the described results. In the current work, we use the same CTPA scan dataset, for a different outcome task. For this task, we trained SANet from scratch using the new label. Considering our highly imbalanced dataset; we used binary cross entropy loss in its weighted version.

For the second baseline model, we used a CTPA-based vision Transformer (ViT) model. ViT model was developed to transfer the success of the self-attention mechanism on NLP tasks into imaging applications^58^. Specifically, we applied Nvidia’s Swin UNETR^59^. Swin UNETR (Shifting windows Unet Transformer) comprises a 3D Swin Transformer hierarchical encoder^47^ and a CNN-based decoder that are connected via skip connections at different resolutions. This model was pre-trained on five different CT datasets. For our classification task, we only use the encoder part of Swin UNETR. In the training process, we first divide the whole scan volume into 3D sub-volumes (tokens) of size 2 $\times$ 2 $\times$ 2. Each token is passed through an embedding layer and concatenated to a positional embedding. The whole scan volume is then partitioned into non-overlapping windows (size 7 $\times$ 7 $\times$ 7) and local self-attention is computed within each window. Finally, two FC layers with 1024 and 64 nodes and the final prediction layer were followed by the encoded embedding layers. We chose this model due to it’s state-of-the-art performance for segmentation task. In addition, using a pre-trained CT dataset model which shares similarity with the target data provided a better starting point than training from scratch.

### Multimodality classification models

We explored different fusion architectures that leveraged information from both the CTPA scans and patient EHRs using different strategies; namely, early (i.e., feature-based), late (i.e., decision-based) and intermediate fusion. The processed data used for the CTPA imaging encoder and the clinical variable encoder were used for our fusion models. All the models shared these same two encoders, one for each data modality, but differed in the information extracted from each encoder and in the fusion technique.

**Figure 7:**
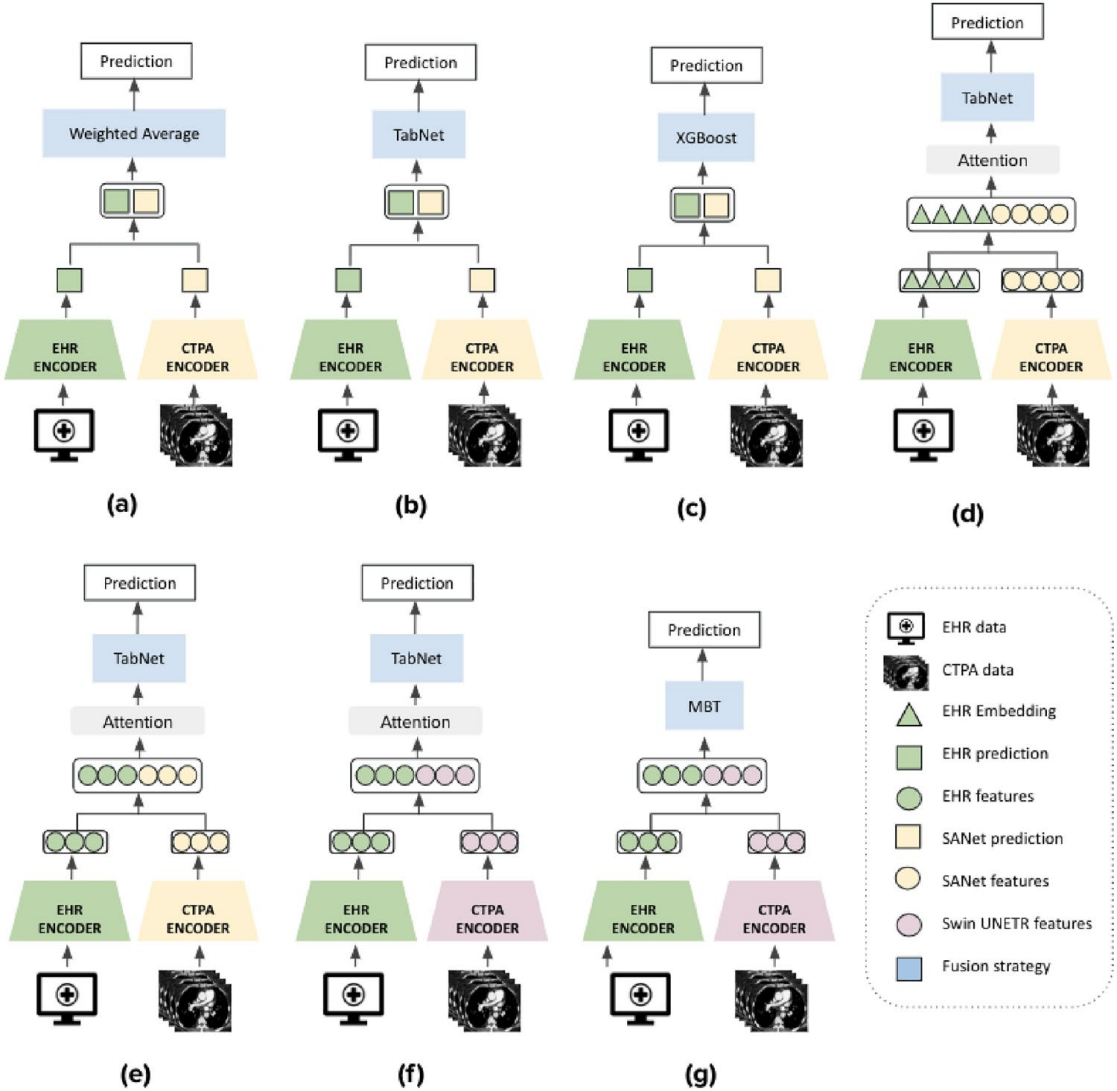
Fusion model architectures: (**a**–**c**)—Late fusion models that differ in the decision model that aggregated the unimodal predictions: weighted average, TabNet and XGBoost respectively. (**d**) Early fusion model that created a shared representation from the concatenation of CTPA features from the SANet and EHR tabular embeddings. The CTPA features are extracted from the last FC layer of SANet. The tabular embeddings are extracted from the TabNet’s Embedding Generator module (Similar to word embeddings in NLP). (**e**) Intermediate fusion model that concatenated extracted latent space features from both SANet and TabNet single modality encoders. The SANet and TabNet features are extracted from the last FC layer of each model. (**f**) This model is identical to (**e**), except for the substitution of the SANet with Swin UNETR model as the CTPA encoder. For (**d**–**f**), the concatenated vector was passed through bilinear attention or dimensionality reduction such as PCA. Finally, the reduced vector was used as input to TabNet for classification. (**g**) The multimodal inputs are encoded by independent TabNet and Swin UNETR Transformer streams and their outputs are concatenated and fused by MBT Transformer encoder.

*Late fusion* extracts the prediction probabilities from each modality separately, and then fuses the results by averaging, majority voting or a meta-classifier based on the predictions from each model. In general, late fusion strategies are much simpler to implement than early or intermediate fusion, particularly when the different modalities vary significantly in terms of data dimensionality and sampling rates. In addition, in this strategy, errors from multiple classifiers tend to be uncorrelated and the method is feature-independent. On the downside, although late fusion can benefit from state-of-the-art models for each modality, usually a simple algorithm at the decision level does not guarantee a full combination of the complementary information^8^. For this work, we used the imaging and clinical data encoders introduced in CTPA imaging encoder and Clinical variable encoder sections, respectively, as our single modality classifiers; specifically, XGBoost and TabNet for the clinical data and SANet for the imaging data. We combined the predictions using either weighted averaging (Fig. 7a) or a learned model: TabNet (Fig. 7b) or XGBoost (Fig. 7c).

In *early fusion* the features of different modalities are first extracted independently and then combined into a single feature vector before feeding into the classifier. This fusion strategy focuses on how best to combine data from multiple, at times very disparate sources, either by removing correlations between modalities or representing the fused data in a lower-dimensional common subspace. The data to be fused are raw or pre-processed and can be joined in many different ways, including concatenation, pooling or by applying a gated unit. In our case, for volumetric image modality, the features needed to be transformed to one-dimension to cope with the EHR data. For our early fusion model, we extracted the image features from the last FC layer of SANet and then concatenated them with the EHR data tabular embeddings using TabNet (Fig. 7d). TabNet contains a custom Embedding Generator module for raw tabular data embedding that can process both categorical and continuous features. The term tabular embedding here refers to the representation of individual tabular features as real-valued vectors in a lower-dimensional space, similar to word embedding in NLP.

In *intermediate fusion* (Fig. 7e, f), a shared representation or fusion layer is constructed that can merge incoming representations of modalities, thereby forcing the network to learn a joint representation of its inputs. The simplest fusion layer is a layer of hidden units, each of which receives input from all modalities. The key difference, compared to early fusion, is that the loss is propagated back to the feature extracting neural networks during training, thus creating better feature representations for each training iteration. Intermediate fusion is implemented with neural networks due to their ability to propagate losses from the prediction model to the feature extraction models. This choice of fusing various representations at different depths is perhaps the most powerful and flexible aspect of deep multimodal fusion as compared to other fusion techniques^60^. For this strategy, we experimented with two different deep learning architectures. The first model utilizes a CNN-based setup where we extracted latent space representations from each modality using SANet and TabNet encoders and combined them using an additional classifier. The latent image and tabular features are extracted from the last FC layer of each model. For our shared representation layer, we used a simple concatenation, followed by dimensionality reduction or attention for multimodal data (Fig. 7e). The second intermediate fusion model (Fig. 7f) is very similar to the previous one. The only difference is that SANet is swapped with the Swin UNETR encoder.

Lastly, in Fig. 7g we apply a novel transformer-based architecture. We implemented a similar model architecture to Multimodal Bottleneck Transformer (MBT)^34^. MBT uses ‘fusion bottlenecks’ for modality fusion at multiple layers. Compared to traditional pairwise self-attention, this model forces information between different modalities to pass through a small number of bottleneck latents, requiring the model to collate and condense relevant information in each modality and share what is necessary. This strategy improves fusion performance and reduces computational cost. Originally, MBT was implemented for audio-visual classification, using an audio and visual transformer based backbones. We revised the model to support our data modalities by using the Swin UNETR and TabNet transformer backbones. The backbones where fused at the layer preceding the last FC layer of each model. After experimenting with the bottleneck hyperparameter we chose 10 as the size of the bottleneck for cross modal attention.

All the representations and embeddings were normalized before being aggregated into the following component. The dimensionality of the two feature components in the shared representation was kept equal to avoid bias towards any single modality. The size of the extracted vector from each modality was 64. The summarized model architectures can be seen in Fig. 7.

### Bilinear attention & dimensionality reduction

For both the CNN based intermediate and early fusion strategies, the shared representation was created by a simple concatenation followed by either a dimensionality reduction algorithm or attention. In general, the common practice is simple concatenation, but we believe this is not enough to utilize inter-modality correlations. The implementation of some form of dimensionality reduction; for example, principal component analysis (PCA), has been shown to improve performance of multimodal fusion by helping the network learn associations between modalities due to distinct underlying distributions^61^. We hypothesized that dimensionality reduction and attention would have a similar effect on the concatenated multimodal vector and would force the selection of the most salient features. We compared the two in Ablation study on architecture choices section. Compared to dimensionality reduction algorithms, attention has certain advantages; namely, because of its differentiability property, attention enables end-to-end training and thus allows better model explainability.

For our framework, we used Bilinear attention network (BAN)^62^ as the attention mechanism. BAN applies attention over bilinear pooling to learn a joint representation between an image and a clinical features. This network was used in many visual question answering (VQA) settings and allows a joint training of an image encoder and clinical data encoder.

### Dataset

**Figure 8:**
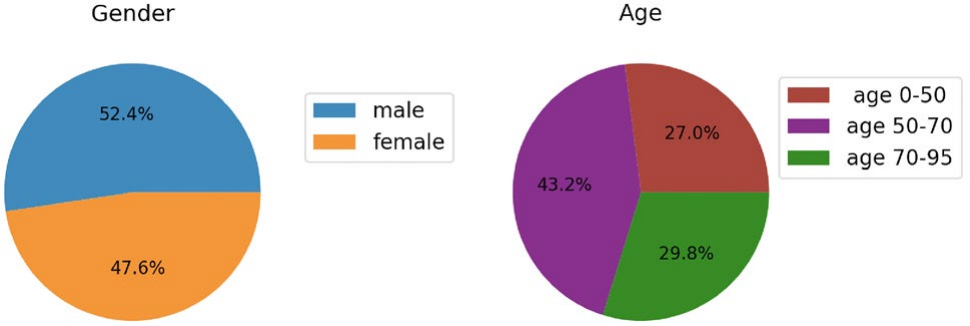
Patients demographics information.

We applied our models to the specific problem of PE risk stratification from CTPA scans and EHR data. This study was performed in line with the principles of the Declaration of Helsinki. A Sheba Medical Center institutional review board (IRB) approval was granted to this retrospective study. The IRB committee of Sheba Medical Center waived the need of informed consent. For cohort selection, we retrieved the data of all patients who underwent CTPA, for whom board certified radiologists had annotated the CTPA reports (report text, time of acquisition). Scans were obtained from the Sheba PACS (Picture Archiving and Communication System) database combined with additional clinical variables and laboratory results collected from the EHR of patients admitted to the Sheba Medical Center. CTPA scans were collected from emergency department (ED) patients who underwent CTPA at Sheba Medical Center from January 2012 to December 2018. Additional epidemiological and clinical data and laboratory results of the patients were collected from the EHR. The detailed characteristics of the collected dataset can be found in Table 4(top). The patient demographics (age and gender) information is presented in Fig. 8. All the CTPA reports were obtained from the Sheba Medical Center RIS (radiology information system) of the Sheba Medical Center. The CTPA scans were anonymized and categorized by expert opinion indicating whether PE existed or not (no severity indication and no marked PE segmentation) according to the radiologist’s report. Each scan was analyzed by a single board certified radiologist, therefore, there were no conflicting labels. The PE severity label (mild /severe PE) was deduced from the single label of 30-day all-cause mortality variable. Selecting one high-level (or series-based) label for the whole 3D scan, with no additional markings or segmentation maps, made this computer-aided diagnosis (CAD) task to be a readily available solution with processing of the scans in a 3D manner.

Our dataset was composed of 358 CTPAs, 38 of whom (10.6%) died within 30 days of the PE diagnosis. We split the dataset into training and testing by year, to ensure no leakage of patient data between the training, validation and testing sets. The model was trained and validated on 2012–2017 data and tested on the held-out year 2018 data. Given the low number of severe PE cases and the resulting class imbalance, we used a stratified five-fold approach to create a cross validation scheme (training and validation). The resulting dataset maintains roughly a 70-10-20 training-validation-test split. The bottom part of Table 4 summarizes the dataset partitioning.

**Table 4:** Dataset specifications.

Category	Numbers and specifications
Imaging	358 CTPA Scans
Laboratory markers	Over 40 tests
Clinical prediction rules	PESI, simplified PESI
Mortality	30-day mortality indication
Patient demographics	Gender, age, cancer history, smoking habits,
	History of heart failure/chronic lung disease,
	Systolic blood pressure, temperature,
	Pulse rate, arterial oxygen saturation
Clinical history	Previous ED visits and hospitalizations,
	Number of days of hospitalization
Inpatient & outpatient medications	641
ICD-9 codes	308
Other	Time (year, month, day, hour),
	Escort type, ED wing, chief complaint

Total (years 2012–2018)	358 (100 %)
Train (years 2012–2017)	242 (67.5 %)
Validation (years 2012–2017)	44 (12.2 %)
Test (year 2018)	72 (20 %)
Severe PE cases	38 (10.6 %)
Severe PE cases in training	23 (9.5%)
Severe PE cases in validation	5 (11.4%)
Severe PE cases in test	10 (13.9 %)

### Statistical analysis

The evaluation of the training included a t-SNE analysis of the extracted features from the dataset. The performance evaluation on the test set covered AUC, sensitivity (also known as recall), specificity, accuracy, the positive predictive value (PPV, also known as precision), and the negative predictive value (NPV). The DeLong method^63^ was used to calculate the 95% confidence intervals for the AUC. The probability thresholds for predicting positive samples were determined by the sensitivities and specificities on the validation set, which ensured high sensitivities while keeping reasonable specificities. These thresholds were determined by Youden’s index^64^, which finds the optimal joint sensitivity and specificity.

### Training and inference

To evaluate the networks fairly, we used the same experimental conditions. We used a batch size of 2 and an Adam optimizer. In order to support our highly imbalanced dataset we used binary cross-entropy loss in its weighted version $L_{bce}$:

1 $$\begin{aligned} L_{bce} = \sum _{i=0}^{N} w_0 y_i \log {x_i} + \sum _{i=0}^{N} w_1 (1-y_i)\log {(1-x_i)} \end{aligned}$$

where $x_i \in (0.0, 1.0)$ represents the predicted probability, and $y_i \in (0,1)$ is the binary ground truth label. $w_i$ where $i \in (0,1)$ are the weights for each class. For additional regularization, a dropout of 0.2 and 0.1 were used in CNN and transformer blocks respectively.

Both the imaging and tabular data modalities were normalized before being fed into each model. We applied minimal data augmentations of random contrast and flipping for the CTPA scans input. We did not want to use other geometric augmentations such as zooming or warping as we did not want to interfere with the networks mortality prediction. Our code was implemented in the Pytorch framework. The network was trained on an NVIDIA 1080Ti GPU.

## Data Availability

The datasets generated and/or analysed during the current study are not publicly available due patient privacy regulations but are available from the corresponding author on reasonable request.

## Code availability

The code generated in this study is available from the corresponding author (N.C.) upon request. All codes related to this project will be made available.
